# A randomized controlled clinical trial of the effects of range of motion exercises and massage on muscle strength in critically ill patients

**DOI:** 10.1186/s13102-022-00489-z

**Published:** 2022-05-26

**Authors:** Elham Rahiminezhad, Mehdi Sadeghi, Mehdi Ahmadinejad, Seyed Iman Mirzadi Gohari, Mahlagha Dehghan

**Affiliations:** 1 Department of Critical Care Nursing, Razi Faculty of Nursing and Midwifery, Kerman University of Medical Sciences, Kerman, Iran; 2 Department of Physical Therapy, Faculty of Allied Medicine, Kerman University of Medical Sciences, Kerman, Iran; 3 Department of Anesthesiology, Shahid Bahonar Hospital, Afzalipour Faculty of Medicine, Kerman University of Medical Sciences, Kerman, Iran; 4 Nursing Research Center, Kerman University of Medical Sciences, Kerman, Iran

**Keywords:** Muscle strength, Range of motion, Massage, Intensive care unit

## Abstract

**Background:**

Atrophy and muscle weakness is a common problem in critically ill patients admitted to the intensive care unit (ICU). Muscle weakness in severe cases can lead to tetraplegia, reduced or lost tendon reflexes, delayed weaning from mechanical ventilation, physical disability, and increased mortality. The aim of this study was to compare the effects of range of motion exercises (ROM) and massage on muscle strength of the patients admitted to ICUs.

**Methods:**

This study was a single-blinded randomized controlled trial conducted in ICUs of Afzalipour hospital in Kerman, southeastern Iran. Ninety conscious ICU patients were randomly divided into three groups (massage, ROM exercises and control). The researcher/co-researcher massaged or did ROM exercises on the patients’ extremities once a day for seven consecutive days. Using a hand-held dynamometer, the co-researcher, rated the muscle strength before, on the fourth and seventh days of intervention at 8 p.m.

**Results:**

The mean muscles strength of the right arm in the ROM exercise and massage groups increased by 0.63 kg, and 0.29 kg, respectively after the intervention compared with before the intervention. The muscle strength of the right arm in the control group reduced by 0.55 kg. The mean muscles strength of the left arm in the ROM exercise and massage groups increased by 0.61 kg and 0.28 kg after the intervention, respectively while it reduced by 0.56 kg in the control group. The mean muscles strength of the right leg in the ROM exercise and massage groups increased by 0.53 kg and 0.27 kg after the intervention compared with before the intervention while it reduced by 0.70 kg in the control group. The mean muscles strength of the left leg in the ROM exercise and massage groups increased by 0.54 kg and 0.26 kg after the intervention compared with before the intervention while it reduced by 0.71 kg in the control group.

**Conclusion:**

The results of the present study showed that ROM exercises and massage were effective interventions in increasing muscle strength of the critically ill patients admitted to intensive care units.

**Supplementary Information:**

The online version contains supplementary material available at 10.1186/s13102-022-00489-z.

## Background

The intensive care unit is where critically ill patients need intensive care for 24 h [1]. Prolonged stays in the intensive care unit often lead to neuromuscular complications, impaired physical function, and impaired mental health [2–5]. Atrophy and muscle weakness are common problems in the ICU. Recent studies show muscle atrophy and loss of muscle mass in mechanically ventilated patients in the early hours of admission [6]. Muscle atrophy occurs due to an imbalance between synthesis and uncontrolled degradation of muscle proteins. In critically ill patients, muscle atrophy is mainly a result of a massive loss of myosin and myoglobin-related proteins in limb and trunk muscles [7]. Long-term treatment of critically ill patients in the intensive care unit reduces hydrostatic pressure in the cardiovascular system, depletes skeletal muscle forces and reduces total energy expenditure, which has negative effects on the musculoskeletal system, cardiovascular system, and circulation and causes muscle weakness [8]. Hyperglycemia, nutritional deprivation, immobilization, sepsis, multiple organ dysfunction syndrome, catecholamines, corticosteroids, and neuromuscular blockers are risk factors for muscle weakness and atrophy [9]. Muscle weakness in severe cases can lead to tetraplegia, reduction or loss of tendon reflex, delay in weaning from mechanical ventilation, and physical disability. Nakanishi et al. showed that upper extremity muscle mass decreased in the ICU during 7 days [8, 10]. They also showed that upper extremity muscle atrophy was associated with in-hospital mortality and physical dysfunction [11]. In addition, lower extremity muscle atrophy and weakness are more common in critically ill patients in the ICU [12]. Puthucheary et al. indicated that rectusfemoris muscle atrophy was rapid after admission to the intensive care unit [12]. Lower extremity muscle atrophy is associated with physical dysfunction and mortality [13–15]. In addition, the study of Thomas et al. showed that even after 12 months of discharge from the intensive care unit, the improvement of physical and cognitive function and quality of life has not been completely achieved [4]. Early intervention in the early hours of admission to the ICU, rehabilitation, early mobilization, respiratory physiotherapy, limb physiotherapy, nutritional support, and electrical muscular stimulation help maintain muscle mass [9]. Therefore, the use of strategies and interventions to reduce such complications in ICU patients seems necessary. Massage therapy has long been known as an essential part of health [16]. It is used in ICUs to reduce physical and mental problems of patients and prevent sympathetic responses [17]. Massage by creating mechanical pressure can relieve muscle spasms, reduce nerve irritation, reduce sympathetic activity, increase blood flow, and increase muscle volume [18, 19]. Swedish massage is the most common type of massage, which is performed on the physical parts of the body (muscles and soft tissues) by using stroking (effleurage), kneading, and vibration movements [20]. Some advantages of Swedish massage are: reduced muscle spasms and pain, improved blood circulation, lymphatic drainage, reduced adhesions in muscles and soft tissues, reduced neuromuscular irritability, release of endorphins, increased intramuscular heating, enhanced immune system, and relaxation [21]. Range of motion exercise is a basic technique used to evaluate and initiate movement in a treatment intervention. ROM exercises include active ROM (AROM), passive ROM (PROM), and active-assistive ROM (A-AROM) [22]. ROM influences all structures taking part in the body’s movement, such as muscles, joint surfaces, ligaments, fascia, arteries, and nerves. ROM exercises also have a positive effect on musculoskeletal health. Some benefits of the ROM exercises include maintaining the mobility of connective and joint tissue, helping blood circulation and vascular dynamics, and strengthening the synovial movement to feed cartilage [23]. Some studies have examined different methods, particularly rehabilitation methods, to prevent muscle weakness and atrophy in critically ill patients admitted to the intensive care unit. Nakanishi et al., Dirks et al., and Maffiuletti et al. showed that neuromuscular electrical stimulation was an effective intervention in preventing muscle atrophy in critically ill patients [24–26]. Santos et al. studied the effects of early rehabilitation using a passive cycle ergometer on the muscle morphology of mechanically ventilated critically ill patients in the intensive care unit. They concluded that early rehabilitation with a passive cycle ergometer could preserve the diaphragm and knee extension morphology of mechanically ventilated patients in the ICU [27]. Brauner et al. showed that a rigorous physical therapy protocol (range of motion exercises, posture change, breathing exercises, active exercises using all limb joints, bed mobility exercises, secretion suction) might facilitate the early rehabilitation of patients with ICU-AW [28]. Verceles et al. demonstrated that compared with routine care, multimodal rehabilitation programs (including muscle strengthening activities, muscle endurance activities, and performance-based aerobic activities) increased muscle strength, mobilization, weaning success, and hospital discharge in patients with ICU-AW [29]. Sarfati et al. showed that passive tilting added to a standard rehabilitation technique did not improve muscle strength in patients admitted to the open heart intensive care unit [30]. Patsaki et al. showed that neuromuscular electrical stimulation and individualized rehabilitation did not lead to further improvement in muscle strength and function of ICU survivors [31]. Therefore, due to the importance of muscle weakness and its consequences in patients admitted to ICUs, it is necessary to use effective interventions to prevent this complication clinically. According to the available evidence, there are contradictory results regarding the effectiveness of various interventions [24–31]. In addition, no study was found to investigate the effect of massage on the muscle strength of patients admitted to the intensive care unit. Therefore, the aim of this study was to compare the effects of ROM exercises and massage on muscle strength of the patients admitted to the intensive care units of Kerman University of Medical Sciences in 2020.

## Method

### Study type, setting, and participants

This single-blinded, controlled, randomized parallel clinical trial was performed on conscious patients admitted to the intensive care units of Afzalipour hospital in Kerman, southeastern Iran. Afzalipour hospital has five intensive care units (medical ICU with 10 beds, surgical ICU with 7 beds, poisoning ICU with 7 beds, COVID-19 ICU 1 with 12 beds, COVID-19 ICU 2 with 7 beds).

Patients aged above 18 years [28], who were on the first day of ICU admission (patients under invasive mechanical ventilation, non-invasive mechanical ventilation, and not mechanically ventilated patients), with a FOUR Score ≥ 14, no amputation [4], no fractures in the lower or upper extremities, no neuromuscular diseases (myasthenia gravis, Guillain–Barre syndrome, botulism and pesticide poisoning) [4], no deep vein thrombosis [32], no skin diseases, no metabolic disorders, including hypokalemia, hypophosphatemia, hypomagnesemia, and no allergy to olive oil in the massage group were eligible to be included in the study. The exclusion criteria were: having been transferred to the ward during the intervention, having any of the disorders listed in the inclusion criteria.

### Data collection tool

A questionnaire was used to collect demographic and background information such as age, sex, type of ward, length of hospital stay before the ICU admission, diagnosis at the time of admission, history of addiction, history of previous illnesses, history of admissions to intensive care units, use of renal replacement therapies, state of consciousness based on FOUR and Glasgow scales, nutritional status, respiratory status, information about device settings (invasive mechanical ventilation, non-invasive mechanical ventilation, high flow), daily medications used by the patient in the ICU, Acute Physiology Age Adjustment Chronic Health Evaluation (APACHE II) and Sequential Organ Failure Assessment (SOFA) scores.

A hand-held dynamometer (Sharif-Exo Model M-201) made in Iran with an error of ± 100 g was used to evaluate muscle strength in this study. Target muscles in the upper extremities include the right and left deltoid, the right and left biceps brachii, and the right and left wrist extensor, while those in the lower extremities include the right and left iliopsoas and rectus femoris, the right and left quadriceps femoris, and the right and left tibialis anterior. Muscle strength was measured three times for each patient, and the maximum score was considered. To determine the reliability of the interarater, the rater, learned how to work with the dynamometer under the supervision of a physiotherapist during three two-hour sessions and performed the evaluation after his approval. In addition, the rater did not play a role in the intervention and random allocation of samples in each group (he was blind). The rater evaluated 15 patients admitted to the ICUs twice (with a two-hour interval). The intraclass correlation coefficients (ICC) for different muscles were more than 0.99 (p < 0.001).

### Procedure

To conduct the research, the researcher first received permission from the hospital management and intensive care units. After obtaining informed written consent, she referred to the ICUs and completed the demographic and background information of the patients who met the inclusion criteria.

#### ROM exercise group

The intervention started on the first day of admission. In addition to routine care, passive, active, and active-assistive ROM exercises were done once a day for seven consecutive days. First, the patient was placed in a supine position, and then the researcher adjusted her position to fit the body mechanics. The ROM exercise area was uncovered and other parts of the body were covered. Passive and active ROM exercises were done according to the patient's condition. The upper extremity ROM exercises include shoulder flexion, shoulder extension, shoulder abduction, elbow flexion and extension, wrist flexion and extension, joints of the thumb and fingers (metacarpophalangeal and interphalangeal joints) and the lower extremity ROM exercises include hip and knee flexion, hip extension, hip abduction, ankle dorsiflexion, plantar flexion. All movements were done rhythmically in ten repetitions. The ROM exercises lasted 30–60 min (Additional file 1). ROM exercises were done from 3 to 7 pm when the workload of the intensive care unit was lower. Indicators of intolerance to ROM exercises include mean arterial pressure ≤ 65 mmHg, systolic blood pressure ≥ 200 mmHg, heart rate ≤ 40 or ≥ 130, oxygen saturation ≤ 88%, respiration rate ≤ 5 or ≥ 30 per minute, and arrhythmia [33]. In the case of intolerance, ROM exercises were postponed until the patient's condition stabilized. In the present study, only two patients developed tachypnea during ROM exercises, so the exercises were done the next day.

The researcher and co-researcher learned ROM exercises under the supervision of a physiotherapist for three sessions (6 h), and they started the intervention after his approval. The co-researcher did ROM exercises for male subjects, and the researcher did them for female subjects.

#### Massage group

The intervention started on the first day of admission. In addition to routine care, the whole body was massaged once a day for seven consecutive days using the Swedish massage style. First, an underpad was used to prevent oil soaking into the patient's mattress. Then, the patient was placed in a supine position with the head at an angle of 30–45 degrees. The massage area was uncovered, and other parts of the body were covered. Then, the whole body was examined for the presence of barriers to massage, including vascular disorders such as DVT, fractures in the lower and upper extremities, skin disease, wounds, infections, and allergies to olive oil. In the case of no problems, Swedish massage was applied, including stroking (effleurage), vibration, and kneading movements. Olive oil (about 20 cc) was used to make the area slippery and easy to massage. The upper extremities, lower extremities, back, and chest were massaged continuously for 30–60 min. The massage was performed from 3 to 7 pm when the workload of the intensive care unit was lower. After the massage, the remaining oil on the patient's body was cleaned with a napkin. The researcher and co-researcher learned how to massage under the supervision of a physiotherapist for three sessions (6 h), and they started the intervention after his approval. The co-researcher massaged the male subjects and the researcher massaged the female subjects [34].

#### Control group

There was no intervention, and they received routine care as usual. A physiotherapist performed routine care in the ICU once a day in the morning, which included respiratory and limb physiotherapy (Additional file 1).

Using a hand-held dynamometer, the co-researcher, who played no role in the intervention and random allocation of the samples, evaluated the muscle strength before (the first day of ICU admission), on the fourth (during intervention) and seventh (after intervention) days of the intervention, as well as on the first (before), fourth (during) and seventh (after) days of admission in the control group at 8:00 p.m.

### Sample size and sampling

**Fig. 1:**
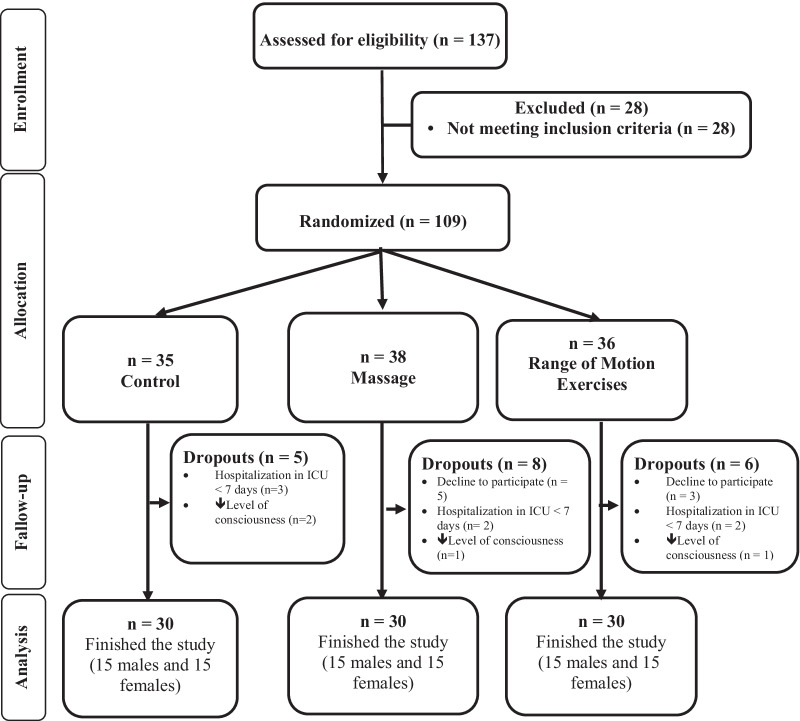
The flow diagram of the study

The samples were selected using the convenience sampling method, and they were allocated into 3 groups by the stratified block randomization method (stratum: gender and age). Labels A, B, or C (A = massage, B = control, and C = ROM exercises) were assigned to the groups, and the block size was 6. The randomization list was generated by using free online software (https://www.sealedenvelope.com/simple-randomiser/v1/lists). The fifth author generated the randomization list, and the first author enrolled the participants and assigned them to the 3 groups. As we did not find a similar study that compared the effects of massage and ROM exercise on muscle strength, we used the rule of thumb, i.e., 30 participants in each group. Power analysis calculations with G*Power software version 3.1.9.2. indicated that (power = 80%, *p* = 0.05, number of groups = 3, and number of measurements = 3) 90 participants would be needed to detect an effect size of 0.275. Totally, 137 samples were assessed for eligibility, of which 109 eligible participants were allocated to the 3 groups. Finally, 30 participants finished the study in each group (Fig. 1). Sampling started in June 2020 and ended in November 2020.

### Statistical analysis

SPSS25 was used for data analysis. A descriptive statistic (frequency, percentage, mean, and standard deviation) was used to describe the patients’ demographic characteristics and clinical history. A mean and standard deviation were used to describe the muscle strength (kg). Chi-square, Fisher's exact, ANOVA and Kruskal–Wallis tests were used to evaluate the similarity of the three groups in terms of study variables. Since the parametric conditions (Shapiro–Wilk test and equality of variances) were fulfilled, repeated measures ANOVA was used to compare the muscle strength (kg) within and between groups before, during, and after the intervention. In addition, the Bonferroni post hoc test was used to compare muscle strength (kg) differences between the three groups at different times. A significance level of < 0.05 was considered.

## Results

### Baseline characteristics of the participants

The mean ages of the samples in the massage, ROM exercises, and control groups were 46.67 ± 17.24, 44.27 ± 13.61, and 47.43 ± 17.36 years, respectively. There was no significant difference between the three groups in terms of ICU type, sex, length of hospital stays before ICU admission, diagnosis, history of addiction, history of underlying disease, history of admission to intensive care units, and history of using renal replacement therapies (p > 0.05) (Table 1). No significant difference was found between the three groups in nutrition, type of diet, respiratory status, and medication regimen (p > 0.05). In addition, the APACHE II score was not significantly different between the three groups before the intervention and Glasgow, FOUR, and SOFA scores were not significantly different between the three groups before, during, and after the intervention (P > 0.05). The GCS in all patients was 15 before, during, and after the intervention.

**Table 1:** Comparison of demographic and clinical characteristics of samples in three groups of massage, ROM exercises, and control

Variable	Group							
	Massage		ROM exercises		Control		Test statistic	P value
	Mean	SD	Mean	SD	Mean	SD		
Age	46.67	17.24	44.27	13.61	47.43	17.36	F = 0.31	0.73

	N	%	N	%	N	%		
*Length of hospital stay before ICU admission*								
1–2 days	29	96.7	27	90.0	26	86.7	χ^2^ = 1.92	0.52
3–4 days	1	3.3	3	10.0	4	13.3		
*Disease diagnosis*							Fisher’s exact test = 10.14	0.19
Respiratory problems	20	71.4	21	77.8	21	70.0		
Pregnancy problems	0	0	2	7.4	2	6.7		
Gastrointestinal problems	3	10.7	3	11.1	0	0		
Poisoning	4	14.3	1	3.7	4	13.3		
Others	1	3.6	0	0	3	10.0		
*Addiction*								
Yes	14	46.7	10	35.7	12	40.0	χ^2^ = 0.73	0.69
No	16	53.3	18	64.3	18	60.0		
*History of chronic disease*								
Yes	18	62.1	13	43.3	18	60.0	χ^2^ = 2.54	0.31
No	11	37.9	17	56.7	12	40.0		
*History of ICU admission*							Fisher’s exact test = 1.49	0.61
Yes	1	3.3	1	3.3	3	10.0		
No	29	96.7	29	96.7	27	90.0		
*Use of renal replacement therapy*							Fisher’s exact test = 1.82	0.77
Yes	1	3.3	0	0	2	6.7		
No	29	96.7	29	100.0	28	93.3		

ROM, Range of motion; SD, Standard deviation

### Main outcome

The mean muscle strength of the right arm in the massage group was 8.49 kg before the intervention, 8.64 kg during the intervention, and 8.78 kg after the intervention. In addition, the mean muscle strength of the right arm of the ROM exercise group was 9.84 kg before the intervention, 10.10 kg during the intervention, and 10.47 kg after the intervention. The mean muscle strength of the control group was 10.89 kg before the intervention, 10.70 kg during the intervention, and 10.34 kg after the intervention. The results of repeated measures ANOVA showed that group-time interaction and time were significant. The results of the Bonferroni post hoc test showed that the pre-intervention score was significantly different between the three groups. In other words, the mean muscle strength of the right arm in the massage group was significantly lower than that of the control group before the intervention (P < 0.001, mean difference = -2.40). Therefore, the difference score was calculated in each group before and after the intervention to control the effect of the variable before the intervention. The mean muscle strength of the right arm in the massage and ROM exercise groups increased by 0.29 kg and 0.63 kg after the intervention compared with before the intervention, while the muscle strength of the right arm in the control group decreased by 0.55 kg. The results of the analysis of variance showed a significant difference between the three groups (P < 0.001, F = 205.54). The Bonferroni post hoc test also showed that changes in the muscle strength of the right arm of the massage group were significantly less than those of the ROM exercise group (P < 0.001, mean difference =—0.34), but significantly higher than those of the control group (P < 0.001, mean difference = 0.84). In addition, muscle strength in the ROM exercise group was significantly higher than that of the control group (P < 0.001, mean difference = 1.18) (Table 2).

**Table 2:** Mean and standard deviation of the right arm muscles strength in three groups of massage, ROM exercises, and control at different times

Right arm muscles strength (kg)	Group					
	Massage		ROM exercises		Control	
	Mean	SD	Mean	SD	Mean	SD
T1	8.49	2.67	9.84	2.07	10.89	2.12
T2	8.64	2.66	10.10	2.06	10.70	2.16
T3	8.78	2.62	10.47	2.09	10.34	2.15
Mean difference (T3–T1)	0.29	0.23	0.63	0.17	− 0.55	0.28

Source of variance	Sum of squares	Degree of freedom	F	P value	Effect size
Time	0.69	1.67	16.94	< 0.001	0.16
Group * time	11.25	3.35	138.03	< 0.001	0.76
Group	195.28	2	6.14	0.003	0.12
Error	1382.42	87			

ROM, Range of motion; SD, Standard deviation; T1, Before intervention; T2, During intervention, T3, After intervention

The mean muscle strength of the left arm in the massage group was 8.35 kg before the intervention, 8.48 kg during the intervention, and 8.63 kg after the intervention. In addition, the mean muscle strength of the left arm of the ROM exercise group was 9.75 kg before the intervention, 9.99 kg during the intervention, and 10.35 kg after the intervention. The mean muscle strength of the left arm in the control group was 10.8 kg before the intervention, 10.58 kg during the intervention, and 10.24 kg after the intervention. The results of the repeated measures ANOVA showed that group-time interaction and time were significant. The results of the Bonferroni post hoc test showed that the pre-intervention score was significantly different between the three groups. In other words, the mean muscle strength of the left arm in the massage group was significantly lower than that of the control group before the intervention (P < 0.001, mean difference = -2.45). Therefore, the difference score was calculated in each group before and after the intervention to control the effect of the variable before the intervention. The mean muscle strength of the left arm in the massage and ROM exercise groups increased by 0.28 kg and 0.61 kg after the intervention compared with before the intervention, while the muscle strength of the left arm in the control group decreased by 0.56 kg. The results of analysis of variance showed a significant difference between the three groups (P < 0.001, F = 173.47). The Bonferroni post hoc test also showed that changes in the muscle strength of the left arm in the massage group were significantly less than those of the ROM exercise group (P < 0.001, mean difference =—0.33), but higher than those of the control group (P < 0.001, mean difference = 0.84). In addition, muscle strength in the ROM exercise group was significantly higher than that of the control group (P < 0.001, mean difference = 1.18) (Table 3).

**Table 3:** Mean and standard deviation of the left arm muscles strength in three groups of massage, ROM exercises and control at different times

Left arm muscles strength (Kg)	Group					
	Massage		ROM exercises		Control	
	Mean	SD	Mean	SD	Mean	SD
T1	8.35	2.65	9.75	2.08	10.80	2.02
T2	8.48	2.61	9.99	2.09	10.58	2.06
T3	8.63	2.56	10.36	2.11	10.24	2.04
Mean difference (T3-T1)	0.28	0.29	0.61	0.17	− 0.57	0.28

Source of variance	Sum of squares	Degree of freedom	F	P value	Effect size
Time	0.55	1.70	11.61	< 0.001	0.12
Group * time	11.26	3.40	119.28	< 0.001	0.73
Group	205.99	2	6.72	0.002	0.13
Error	1333.16	87			

ROM, Range of motion; SD, Standard deviation; T1, Before intervention; T2, During intervention; T3, After intervention

The mean muscle strength of the right leg in the massage group was 10.86 kg before the intervention, 11.00 kg during the intervention, and 11.14 kg after the intervention. In addition, the mean muscle strength of the right leg in the ROM exercise group was 11.70 kg before the intervention, 11.97 kg during the intervention, and 12.23 kg after the intervention. The mean muscle strength of the right leg in the control group was 11.46 kg before the intervention, 11.09 kg during the intervention, and 10.76 kg after the intervention. The results of repeated measures ANOVA showed that group-time interaction and time were significant. In other words, the muscle strength of the right leg increased significantly in the massage and ROM exercise groups, while it decreased significantly in the control group. The pre- and post-intervention difference scores were calculated in each group. The mean muscle strength of the right leg in the massage and ROM exercise groups increased by 0.27 kg and 0.53 kg after the intervention compared with before the intervention, while the muscle strength of the right leg in the control group decreased by 0.70 kg. The results of the analysis of variance showed a significant difference between the three groups (P < 0.001, F = 204.04). The Bonferroni post hoc test showed that the changes in the muscle strength of the right leg in the massage group were significantly lower than those of the ROM exercise group (P < 0.001, mean difference =—0.25), but higher than those of the control group (P < 0.001, mean difference = 0.97). In addition, muscle strength in the ROM exercise group was significantly higher than that of the control group (P < 0.001, mean difference = 1.22) (Table 4).

**Table 4:** Mean and standard deviation of the right leg muscles strength in three groups of massage, ROM exercises and control at different times

Right leg muscles strength (Kg)	Group					
	Massage		ROM exercise		Control	
	Mean	SD	Mean	SD	Mean	SD
T1	10.86	2.04	11.70	1.49	11.46	1.81
T2	11.00	2.06	11.97	1.46	11.09	1.98
T3	11.14	2.03	12.23	1.57	10.76	2.02
Mean difference (T3-T1)	0.27	0.18	0.53	0.21	− 0.70	0.33

Source of variance	Sum of squares	Degree of freedom	F	P value	Effect size
Time	0.06	2	1.01	0.37	0.01
Group * time	12.50	4	115.74	< 0.001	0.73
Group	50.96	2	2.51	0.09	0.06
Error	882.13	87			

ROM, Range of motion; SD, Standard deviation; T1, Before intervention; T2, During intervention; T3, After intervention

The mean muscle strength of the left leg in the massage group was 10.82 kg before the intervention, 10.93 kg during the intervention, and 11.08 kg after the intervention. In addition, the mean muscle strength of the left leg of the ROM exercise group was 11.64 kg before the intervention, 11.89 kg during the intervention, and 12.18 kg after the intervention. The mean muscle strength of the left leg in the control group was 11.42 kg before the intervention, 11.01 kg during the intervention, and 10.70 kg after the intervention. The results of repeated measures ANOVA showed that group-time interaction and time were significant. In other words, the muscle strength of the left leg increased significantly in the massage and ROM exercise groups, while it decreased significantly in the control group. The pre- and post-intervention difference scores were calculated in each group. The mean muscle strength of the left leg in the massage and ROM exercise groups increased by 0.26 kg and 0.54 kg after the intervention compared with before the intervention, while the muscle strength of the left leg in the control group decreased by 0.71 kg. The results of the analysis of variance showed a significant difference between the three groups (P < 0.001, F = 241.12). The Bonferroni post hoc test showed that the changes in the muscle strength of the left leg in the massage group were significantly lower than those of the ROM exercise group (P < 0.001, mean difference = -0.28), but higher than those of the control group (P < 0.001, mean difference = 0.97). In addition, muscle strength in the ROM exercise group was significantly higher than that of the control group (P < 0.001, mean difference = 1.25) (Table 5).

**Table 5:** Mean and standard deviation of the left leg muscles strength in three groups of massage, ROM exercises and control at different times

Left leg muscles strength (kg)	Group					
	Massage		ROM exercise		Control	
	Mean	SD	Mean	SD	Mean	SD
T1	10.82	2.06	11.64	1.52	11.42	1.78
T2	10.93	2.08	11.89	1.49	11.01	1.93
T3	11.08	2.05	12.18	1.59	10.70	1.96
Mean difference (T3–T1)	0.26	0.20	0.54	0.19	− 0.71	0.29

Source of variance	Sum of squares	Degree of freedom	F	P value	Effect size
Time	0.08	2	1.73	0.18	0.02
Group * time	12.94	4	133.74	< 0.001	0.76
Group	49.83	2	2.46	0.09	0.05
Error	881.98	87			

ROM, Range of motion; SD, Standard deviation; T1, Before intervention; T2, During intervention; T3, After intervention

### Adverse events

Only two samples in the ROM exercises group experienced tachypnea during one session of the intervention. We did not observe any particular side effects during the massage sessions.

## Discussion

### Limitations

The present study had some limitations. The sample size was relatively small. Although 30 participants in each group completed the study, future studies with larger sample sizes may better confirm the results of the present study. Since the patients admitted to the ICU were not in good condition and had many physical, mental, and psychological changes, some of the patients were reluctant to participate in the study. Future studies can further confirm the results of the present study by measuring muscle activity with electromyography.

The results of the present study showed that the muscle strength of the right and left arms, the right and left legs in the ROM exercise and massage groups increased after the intervention compared with before the intervention, while the muscle strength of all upper and lower extremities decreased significantly in the control group. The results showed that ROM exercises and massage had an effect on the muscle strength of the patients admitted to the intensive care unit, respectively.

Verceles et al. showed that compared with routine care, a multimodal rehabilitation program (including muscle-strengthening activities, muscle endurance activities, and performance-based aerobic activities) increased muscle strength, mobilization, weaning success, and hospital discharge in patients with ICU-AW [29]. Brauner et al. showed that an intense physiotherapy protocol (ROM exercises, posture change, breathing exercises, active exercises using all limb joints, bed mobility exercises, secretion suction) might facilitate the early rehabilitation in patients with ICU-AW [28]. Anekwe et al. in a systematic review and meta-analysis showed that early rehabilitation was associated with a decreased likelihood of developing ICU-AW [35]. Veldema et al. showed that cycle ergometer training and resistance training improved lower limb muscle strength and the ability to walk in ICU-AW patients [36]. Hosseini et al. showed that passive range of motion exercises significantly improved motor function in both the upper and lower limbs of people with strokes [37]. Nakanishi et al. showed that neuromuscular electrical stimulation prevented upper and lower extremity muscle atrophy in critically ill patients [26]. Nakamura et al. showed that skeletal muscle electrical stimulation has the potential to inhibit muscle volume loss in critical care [38]. Studies mentioned above support the present study because electrical muscular stimulation, exercise, and ROM exercises were used to increase muscle strength and manage muscle weakness. ROM exercises affect muscles, the joint surface, the ligaments, the fascia, the arteries, and the nerves, leading to the mobilization of the joint, soft tissue, and muscles, minimizing the loss of tissue flexibility, and increasing muscle strength and hypertrophy. They also increase synovial fluid lubrication of the joint and thus increase the rate of intra-articular cartilage healing and regeneration [23, 39]. Early mobilization reduces the duration of inactivity, improves venous return, increases the amount of oxygen distributed to tissues, and improves muscle dysfunction [33–35, 39].

In contrast, Sarfati et al. studied the effectiveness of early passive tilting in minimizing ICU-AW in patients admitted to the intensive care unit. The results showed that passive tilting added to a standard rehabilitation method did not improve muscle strength in patients admitted to the open heart intensive care unit [30]. Patsaki et al. showed that neuromuscular stimulation and individualized rehabilitation did not lead to further improvement in muscle strength and function of ICU survivors [31]. The results of Sarfati et al., and Patsaki et al. were not consistent with those of the present study because the study of Sarfati et al. was different from the present study in the type of intervention and the study setting. The samples in the study by Patsaki et al. were ICU survivors, and neuromuscular electrical stimulation was done on their lower extremities, but the samples in the present study were ICU patients who underwent intervention and early rehabilitation. Massage and ROM exercises were done on the upper and lower extremities of the patients admitted to intensive care units (surgical, poisoning, medical, and COVID-19).

No study evaluated the effect of massage on muscle strength in patients admitted to the intensive care unit. Massage stimulates the lymphatic system and increases blood circulation, so more oxygen reaches the organs, and because of the applied pressure, the amount of blood exchanged between the tissues increases. Massage also improves the elasticity of the muscle fibers and helps the muscle contract by increasing the permeability of capillaries and muscle tissue. Musculoskeletal weakness, atrophy, and physical disability are common in critically ill patients in intensive care units, and a significant reduction in muscle mass begins within 3 days of ICU admission and then gradually worsens [10, 26]. Long hospital stays, physical disability, and increased mortality are all consequences of decreased muscle strength and mass [40]. It is therefore essential to use effective interventions to reduce the incidence of this disorder in these patients. Massage therapy and ROM exercises can be used to maintain muscle strength. It's important to note that no study has looked at how massage affects muscle strength, so more research is needed to get better and more accurate results from it (Additional file 2).

## Conclusion

The results of the present study showed that ROM exercises and massage could have a significant effect on the muscle strength of patients admitted to intensive care units. Owing to the fact that patients admitted to intensive care units are generally immobile for a long time and their muscles become weak over time, these treatments can have a significant impact on the muscle strength of patients admitted to intensive care units. It is suggested that ICU nurses implement these treatments in the routine care of patients and do ROM exercises in intensive care units more carefully and regularly. Range of motion exercises and massage therapy can be used in home nursing care and can be taught to the patients’ caregivers for post intensive care discharge to prevent and improve ICU-AW. It may also be effective in improving muscle strength and relaxation for other patients with long hospital stays. Future studies are suggested to evaluate the effects of massage and ROM exercises on quality of life, the strength of respiratory muscles, length of hospital stay, and post-intensive care syndrome in patients with ICU-AW.

## Supplementary Information

**Additional file 1**. Comparison of range of motion exercises as an intervention with routine care.

**Additional file 2**. Clinical trial protocol Iranian registry of clinical trials.

## Data Availability

The datasets used for the current study are available from the corresponding author upon request.
