# Supplementary material for: A randomized controlled clinical trial of the effects of range of motion exercises and massage on muscle strength in critically ill patients
Source: BMC Sports Sci Med Rehabil. 2022 May 26;14:96. doi: 10.1186/s13102-022-00489-z (PMC9134983; doi:10.1186/s13102-022-00489-z)
Supplement: Supplementary file 1 — Additional file 1. Comparison of range of motion exercises as an intervention with routine care. [file 13102_2022_489_MOESM1_ESM.docx]

**Supplementary. Comparison of range of motion exercises as an intervention with routine care**

|  | | Intervention group | | Routine care | |
| --- | --- | --- | --- | --- | --- |
|  |  | Passive, Active, Active-Assistive | | Passive, Active, Active-Assistive | |
| Range of motion exercises (ROM) | Upper  extremity | 10  repetitions | Shoulder  Flexion/ extension/ abduction | 10  repetitions | Shoulder  Flexion |
|  |  |  | Elbow  flexion/extension |  | Elbow  flexion/extension |
|  |  |  | Wrist  flexion/extension |  | No |
|  |  |  | Joints of the Thumb and Fingers (metacarpophalangeal  and interphalangeal joints (  Flexion/ Extension/Abduction /Adduction |  | No |
|  | Lower  extremity |  | Hip  flexion/abduction/adduction |  | Hip  flexion |
|  |  |  | Knee flexion |  | Knee flexion |
|  |  |  | Ankle dorsiflexion/  plantar flexion |  | plantar flexion |
|  | Total time |  | 30 -60 minutes |  | 15 minutes |
