# Supplementary material for: A randomized controlled clinical trial of the effects of range of motion exercises and massage on muscle strength in critically ill patients
Source: BMC Sports Sci Med Rehabil. 2022 May 26;14:96. doi: 10.1186/s13102-022-00489-z (PMC9134983; doi:10.1186/s13102-022-00489-z)
Supplement: Supplementary file 2 — Additional file 2. Clinical trial protocol Iranian registry of clinical trials. [file 13102_2022_489_MOESM2_ESM.pdf]

Clinical Trial Protocol
Iranian Registry of Clinical Trials

02 Apr 2022

Comparison of the Effect of Massage and Range of Motion Exercises on Intensive Care Units Acquired Weakness

Protocol summary

Study aim

Comparison of the Effect of massage and range of motion exercises on intensive care unit acquired weakness in hospitals affiliated to Kerman University of Medical Sciences, 2019

Design

A clinical trial with two intervention groups with parallel groups, without a control group, without blinding, randomized

Settings and conduct

The study will be done as an interventional study in two groups of massage and range of motion exercises in two ICUs in two hospitals.

Participants/Inclusion and exclusion criteria

The study sample consists of all patients admitted to the intensive care unit with inclusion criteria. Inclusion criteria: First day ICU patients (patients undergoing invasive mechanical ventilation, non-invasive mechanical ventilation, and patients not under mechanical ventilation), FOUR Score in patients not mechanically ventilated (ER 4, MR 4, CR 4, R 4) and in patients undergoing mechanical ventilation (ER 4, MR 4, CR 4, R 1). Exclusion criteria: Having amputation, upper and lower extremity fractures, neuromuscular diseases (myasthenia gravis, guillotine, botulism, and pesticide poisoning, etc.), deep vein thrombosis, skin diseases, metabolic disorders: hypocalcemia, hypophosphatemia, hypomagnesemia, and an allergy to olive oil in the massage group.

Intervention groups

Massage group: The intervention in this group begins on the first day of admission. In addition to providing routine care, the researcher massages the entire body once a day for 7 consecutive days using the Swedish massage style. Range of motion exercises group: The intervention in this group begins on the first day of admission. In addition to providing routine care, the researcher performs a passive and active range of motion exercises once a day for 7 consecutive days.

Main outcome variables

Change in the score of Intensive Care Units Acquired Weakness

General information

Reason for update

Acronym

IRCT registration information

IRCT registration number: **IRCT20200203046358N1**
Registration date: **2020-02-14, 1398/11/25**
Registration timing: **prospective**

Last update: **2020-02-14, 1398/11/25**
Update count: **0**

Registration date

2020-02-14, 1398/11/25

Registrant information

Name

Elham Rahiminezhad

Name of organization / entity

Country

Iran (Islamic Republic of)

Phone

+98 34 3251 2840

Email address

e.rahimi@kmu.ac.ir

Recruitment status

Recruitment complete

Funding source

Expected recruitment start date

2020-02-20, 1398/12/01

Expected recruitment end date

2020-07-22, 1399/05/01

Actual recruitment start date

empty

Actual recruitment end date

empty

Trial completion date
empty

Scientific title
Comparison of the Effect of Massage and Range of Motion Exercises on Intensive Care Units Acquired Weakness

Public title
Comparison of the Effect of Massage and Range of Motion Exercises on Intensive Care Units Acquired Weakness

Purpose
Supportive

Inclusion/Exclusion criteria
Inclusion criteria:
First day ICU patients (patients undergoing invasive mechanical ventilation, non-invasive mechanical ventilation, and patients not under mechanical ventilation) FOUR Score in patients not mechanically ventilated (eye response score 1, motor response 2, corneal reflex 2, respiration 1) and in patients undergoing mechanical ventilation (eye response score 2, motor response 2, corneal reflex 2, respiration 1)
Exclusion criteria:
Having amputation Having upper and lower extremity fractures Having neuromuscular diseases (myasthenia gravis, guillotine, botulism, and pesticide poisoning, etc.) Having deep vein thrombosis Having skin diseases Having metabolic disorders: hypocalcemia, hypophosphatemia, hypomagnesemia Having allergy to olive oil in the massage group

Age
From **18 years** old

Gender
Both

Phase
N/A

Groups that have been masked
No information

Sample size
Target sample size: **10**

Randomization (investigator's opinion)
Randomized

Randomization description
Samples will be divided into two groups (massage group, range of motion exercises) using the minimization method. In the minimization method, sex, age (±2), and hospital are considered as matched variables at baseline. In other words, in each hospital, the first sample will be placed in one of the groups using the lottery, and the next sample will be randomly assigned to one of the groups after matching the groups by gender and age. This process will continue until the sample volume is complete. Random allocation of samples to each group will be done by a researcher who has no role in intervention and outcome measurement.

Blinding (investigator's opinion)
Not blinded

Blinding description

Placebo
Not used

Assignment
Parallel

Other design features

Secondary Ids

empty

Ethics committees

1

Ethics committee
Name of ethics committee
Ethics committee of Kerman University of Medical Sciences
Street address
Kerman University of Medical Sciences,Medical University Campus,Haft-Bagh Highway, Kerman
City
Kerman
Province
Kerman
Postal code
7616913555

Approval date
2020-01-20, 1398/10/30

Ethics committee reference number
IR.KMU.REC.1398.558

Health conditions studied

1

Description of health condition studied
Intensive Care Units Acquired Weakness

ICD-10 code

ICD-10 code description

Primary outcomes

1

Description
Change in the score of Intensive Care Units Acquired Weakness

Timepoint
Before the intervention, the fourth day of the intervention, and at the end of the seventh day of the intervention

Method of measurement
The Medical Research Council (MRC) physical strength examination and the Lafayette Hand-Held Dynamometer (HHD) Model (01163)

Secondary outcomes

empty

Intervention groups

1

Description

Intervention group: Massage group: Intervention in this group begins on the first day of admission. In this group, in addition to providing routine care, the researcher/fellow researcher massages the entire body once a day for 7consecutive days using the Swedish massage style. To do the massage, first placing the mat so that the patient's mattress will not get oily and the patient is positioned. Massage is in such a way that the patient will be in the supine position and the head is positioned at an angle of 30 to 45 degrees. Swedish massage comprises strokes, gliding, and vibrating. For massage, olive oil is used to the extent that facilitates massage (approx. 20 ccs). The whole-body massage will be continuous and lasts 30-60 minutes. Massage is performed at 3 to 7 pm when the intensive care unit workload is less. After the massage is completed, the patient's entire body is cleaned with a napkin to cleanse the remaining oil on the patient's body.

Category

Rehabilitation

2

Description

Intervention group: Range of motion exercises group: The intervention in this group begins on the first day of admission. In this group, in addition to providing routine care, the researcher performs a passive and active range of motion exercises once a day for 7 consecutive days. The intervention is that the patient is first placed in the supine position. Passive range of motion exercises of the upper extremities include fingers, wrists, elbows, shoulders, and lower extremity passive range of motion exercises include toes, ankles, knees, thighs. All movements are performed gently and rhythmically and repeated 10 times. The range of motion exercises will take 30-60 minutes. The parameters indicating intolerance of range of motion exercises are as follows: mean arterial pressure ≤65 mmHg, systolic blood pressure 200≤ mmHg, heart rate ≤40 or 130≤, oxygen saturation percentage ≤88%, respiratory rate ≤5 or 30≤ per minute, and arrhythmia. In case of intolerance, until the patient's condition is stabilized, the continuation of the range of motion exercises will be postponed. The range of motion exercises will be performed from 3 to 7 pm.

Category

Rehabilitation

Recruitment centers

1

Recruitment center

Name of recruitment center

Shahid Bahonar Hospital

Full name of responsible person

Mahdi Ahmadinejad

Street address

Gharani street

City

Kerman

Province

Kerman

Postal code

7613747181

Phone

+98 34 3223 5011

Email

Ahmadinejadmahdi@yahoo.com

2

Recruitment center

Name of recruitment center

Afzalipour Hospital

Full name of responsible person

Mehdi Hayatbakhsh Abbasi

Street address

Imam Khomeini Highway, Next to Shahid Bahonar University, Afzalipour Hospital

City

Kerman

Province

Kerman

Postal code

۷۶۱۶۹۱۳۹۱۱

Phone

+98 34 3132 8000

Email

m24672@yahoo.com

Sponsors / Funding sources

1

Sponsor

Name of organization / entity

Kerman University of Medical Sciences

Full name of responsible person

Abbas Pardakhti

Street address

Kerman University of Medical Sciences,Medical University Campus,Haft-Bagh Highway, Kerman, Iran

City

Kerman

Province

Kerman

Postal code

7616913555

Phone

+98 34 3226 3855

Email

vcr@kmu.ac.ir

Grant name

Grant code / Reference number

Is the source of funding the same sponsor organization/entity?

Yes

Title of funding source
Kerman University of Medical Sciences
Proportion provided by this source
100
Public or private sector
Public
Domestic or foreign origin
Domestic
Category of foreign source of funding
empty
Country of origin
Type of organization providing the funding
Academic

Person responsible for general inquiries

Contact
Name of organization / entity
Kerman University of Medical Sciences
Full name of responsible person
Elham Rahiminezhad
Position
M.Sc. Nursing Student
Latest degree
Bachelor
Other areas of specialty/work
Nursery
Street address
Kerman University of Medical Sciences,Medical University Campus,Haft-Bagh Highway, Kerman, Iran
City
Kerman
Province
Kerman
Postal code
7616913555
Phone
+98 34 3251 2840
Email
elham.rahiminezhad@yahoo.com

Person responsible for scientific inquiries

Contact
Name of organization / entity
Kerman University of Medical Sciences
Full name of responsible person
Elham Rahiminezhad
Position
M.Sc. Nursing Student
Latest degree
Bachelor
Other areas of specialty/work
Nursery
Street address
Kerman University of Medical Sciences,Medical University Campus,Haft-Bagh Highway, Kerman
City
Kerman
Province
Kerman
Postal code
7616913555
Phone
+98 34 3251 2840
Email
elham.rahiminezhad @yahoo.com

Person responsible for updating data

Contact
Name of organization / entity
Kerman University of Medical Sciences
Full name of responsible person
Elham Rahiminezhad
Position
M.Sc. Nursing Student
Latest degree
Bachelor
Other areas of specialty/work
Nursery
Street address
Kerman University of Medical Sciences,Medical University Campus,Haft-Bagh Highway, Kerman, Iran
City
Kerman
Province
Kerman
Postal code
7616913555
Phone
+98 34 3251 2840
Email
elham.rahiminezhad @yahoo.com

Sharing plan

Deidentified Individual Participant Data Set (IPD)
Undecided - It is not yet known if there will be a plan to make this available
Study Protocol
Undecided - It is not yet known if there will be a plan to make this available
Statistical Analysis Plan
Undecided - It is not yet known if there will be a plan to make this available
Informed Consent Form
Undecided - It is not yet known if there will be a plan to make this available
Clinical Study Report
Undecided - It is not yet known if there will be a plan to make this available
Analytic Code
Undecided - It is not yet known if there will be a plan to make this available
Data Dictionary
Undecided - It is not yet known if there will be a plan to make this available
